# Milk-Sickness

**Published:** 1882-04

**Authors:** K. H. Davis

**Affiliations:** McElmore’s Cove, Ga.


### MILK-SICKNESS.

BY K. H. DAVIS, M.D., McELMORE'S COVE, GA.

*An Inaugural Thesis Presented to the Faculty of the Atlanta Medical College.*

Milk-sickness, milk-poison or trembles, as it is frequently called by the illiterate, is the subject selected for this thesis, because I have frequently been called upon to treat the disease, and after examining all the text-books at my command, I could find nothing on the subject that gave a satisfactory description of this terrible disorder, or any suggestions respecting its treatment. Milk-sickness or milk-poisoning is caused by some unknown toxic substance that herbiverous animals eat when grazing or feeding on the wild vegetables that grow upon the rich woody hillsides and about coves of the mountainous sections of the United States. The disorder is usually observed in the horse and cow; it possibly occurs also in other animals. The animals that are kept in a stable or in a dry lot until after the morning dew dries off the grass and shrubs, are never poisoned in this way; they always escape, while animals that feed on vegetable growths while wet with dew, are nearly certain to be poisoned. During rainy or damp weather the same thing befalls them. This can be explained as follows: Animals eat more greedily when the vegetation is saturated with water than they do when the vegetation is dry. Their instinct admonishes them to be more cautious when they do not feed so voraciously. There is a common idea among uneducated people that the disease is caused by some mineral substance or by a gas rising out of the earth during the night. They will tell you that they have spread out hay or fodder on the ground in the suspected localities, and then fed cattle with it, producing the disease under consideration. I have no doubt of the sincerity of their belief, and if the experiment was verifiable, it would go a long way toward settling the question. I think this opinion, however, is erroneous. Experiments conducted by ignorant men, with preconceived opinions, caused by the teachings of their ancestors and contended for by friends, neighbors and acquaintances, are not entitled to much credit unless they are supported by a great amount of trustworthy evidence. All persons of intelligence in the localities invaded by this disease are agreed that clearing and tilling the land where the poison exists destroys it, and affords complete protection against its deadly influence. I cannot understand how clearing and cultivating the land would remove a mineral poison from the earth. Other observers, with more reason, hold to the idea that the poison is a vegetable product, that the vegetable is of a delicate nature, and that it dies out from the effect of the sunshine or is eradicated by cultivation. That the weed, bush or vine which furnishes the poison could be destroyed in that manner appears more reasonable. Some investigators have gone so far as to affirm that the three-leaved Rhus vine is the vegetable that supplies this poison. They give as a reason for this belief that where there is no Rhus vine there is never any milk-sickness found. I think we have good reason for rejecting the Rhus-vine theory, because in Rhus vine poisoning there is a vesicular eruption on the skin and mucous membrane of the mouth, tongue, fauces, throat, and frequently throughout the whole alimentary canal. There is no phenomena of this kind observed in milk-sickness. From what I have seen and learned of this disease, the cause is very obscure and as yet unsettled. Animals may and do have the poison in their systems, and yet go about and appear fat and sleek as if they were in the best of health until they become fatigued or heated by violent exercise; they will then begin to tremble and stagger, and finally fall down to rise no more. They appear to suffer the most intense pain for a short time and die. The people in the infected districts very o ten chase their beef cattle around with dogs until they are heated by the exercise; then if the cattle do not show any signs of the disease they are killed and eaten as beef without fear. When any animal dies of milk-sick poison, the flesh possesses toxic qualities—poisoning any one who ingests it. Dogs, wolves, buzzards and hogs are frequently killed in this manner. Cows that are giving milk do not often die of the mild-sick poison. The poison is said to pass out of the system through the lacteal glands, but the milk kills the calf that sucks it, or anything else that may drink it is liable to be poisoned, while the cow that furnished the milk will escape the disease and show no signs of being sick.

Milk-sickness in the human subject is always caused by drinking infected milk, eating infected beef, butter, or cheese. Sweet milk or cream is said to be the most pernicious. It is claimed that milk churned (butter milk) is not poisonous whilst the butter which is made from the milk is sure to contain the poison in large quantities. It is the general opinion that the poison atoms get concentrated in certain particles of butter or cream. This may or may not be true. I think it is getting the question down to rather a fine point. Frequently a whole family will eat a meal at the same table, all partaking of the same diet, using milk and butter out of the same vessel, and only two or three out of the whole number may be attacked very soon—all the others escaping. I have frequently witnes-ed such instances. But occasionally a whole family will be attacked at once, soon after partaking of a meal.

The following are the symptoms of the disease in the human subject: Very soon after eating a meal containing the infected beef, butter, milk, or cheese the patient begins to feel nauseated or "sick at the stomach." There is an acute, fixed pain in the epigastrium with a sense of burning heat; extreme soreness, increased by pressure, even the weight of the bed cover is painful; deglutition and the movements of respiration embarrassed; frequent vomiting attended with increased pain; hiccough; sudden and very great prostration of strength; a rapid, small, wiry pulse; great restlessnesss; extreme anxiety; intense thirst for cold drinks; the tongue is red around the edges and sharp at the point; bowels obstinately constipated; the fever runs pretty high—I cannot give the exact temperature, as I have never tested it with the clinical thermometer; the mind remains clear to the last, so far as I have observed. The extreme nausea is the worst system of all. It is actually painful to witness the terrible and continued efforts at vomiting; then we find pain in the head, and back, and a constant tremulous motion of the hands arms and legs and a peculiar odor of the breath, compared to the odor of a young calf by the common people (perhaps this is imaginary); the bowels soon become tympanitic, sensitive and acutely painful. Post mortem examination shows signs of inflammation in the stomach and extending throughout the entire alimentary tract, and the mesentery is intensely engorged. In the case of Miss———who died in Walker county, the post mortem being performed by Dr. Green B. Gordon, the opposing internal surfaces of the small intestine at one place was united by adhesive inflammation for three or four inches of its length, making it impossible to procure any action on the bowels.

When a patient does recover from an attack of milk-sickness the deviation from the standard of health is so great that complete recovery is not attained. He remains tremulous and is easily fatigued and is of "no account for anything."

The prognosis is always doubtful, unless proper treatment is used at an early stage of the disease. If properly treated, most of the cases recover. The diagnosis is frequently attended with difficulty. The diseases most likely to be mistaken for it are acute gastritis, or malarial fever of a remittent type, complicated with gastric disturbance. The first two cases of milk-sickness I ever encountered I saw in consultation with another physician, and mistook the disease for malarial fever, with gastric complications. The usual treatment for malarial fever was used. We gave calomel, followed up with quinine and Dover's powder in decided doses. This ought to have relieved malarial fever. Both patients died promptly.

The only method of making a proper diagnosis is to obtain a careful history of the whole case with all of its surroundings. If the patient was in good health and was suddenly attacked soon after eating butter, milk or beef, and if milk-sickness is known to have been in the locality before, we may be greatly aided in arriving at a correct diagnosis. If a genuine case has ever been seen before, you are certain to recognize it afterwards. There is a peculiarity of expression about it which is not likely to be forgotten.

*Treatment.*—The first indications are to open the bowels *thoroughly* and *freely*. It is an axiom among the people in the infected districts, that if a thorough action on the bowels can be secured the patient will be almost certain to recover. This corresponds with my own observation. The next thing to be done is to administer alcoholic stimulants in combination with olive oil. This treatment is empiric, I know that, but we use it because it cures, without being able to give any other reason. The constipation is very obstinate and is difficult to overcome, and on account of the extreme irritability of the stomach the administration of remedies is seriously impeded, but by persistent use of cathartics, aided by rectal injections, we will usually succeed. In one case, after these means had all failed, we immersed the patient in a barrel of hot water up to her neck, as hot as she could bear it, and this effected relaxation, and produced the desired result. Frequently about the time the patient begins to convalesce they become jaundiced, the eyes and skin are "as yellow as a pumpkin." Under these circumstances a few old-fashioned doses of calomel bring about a cure surely and quickly. I will repeat here what I firmly believe, that whisky and sweet oil, in doses of one tablespoonful of each every two hours, is the king of remedies for milk-sickness.

